# Correction: The signal pathways and treatment of cytokine storm in COVID-19

**DOI:** 10.1038/s41392-021-00744-8

**Published:** 2021-08-31

**Authors:** Lan Yang, Xueru Xie, Zikun Tu, Jinrong Fu, Damo Xu, Yufeng Zhou

**Affiliations:** 1 Institute of Pediatrics, Children’s Hospital of Fudan University, National Children’s Medical Center, and the Shanghai Key Laboratory of Medical Epigenetics, International Co-laboratory of Medical Epigenetics and Metabolism, Ministry of Science and Technology, Institutes of Biomedical Sciences, Fudan University, Shanghai, China; 2 National Health Commission (NHC) Key Laboratory of Neonatal Diseases, Fudan University, Shanghai, China; 3 General Department, Children’s Hospital of Fudan University, Shanghai, China; 4 State Key Laboratory of Respiratory Disease for Allergy at Shenzhen University, Shenzhen Key Laboratory of Allergy and Immunology, Shenzhen University School of Medicine, Shenzhen, China; 5 The Affiliated Luohu Hospital of Shenzhen University, Shenzhen Luohu Hospital Group, Shenzhen, China

**Keywords:** Infectious diseases, Infectious diseases

Correction to: *Signal Transduction and Targeted Therapy* 10.1038/s41392-021-00679-0, published online 7 July 2021

After online publication of the article,^1^ the editor noticed that Figs. 1 and 4 were produced with the assistance of Servier Medical Art (https://smart.servier.com) without citation. The authors requested to add a statement in the Acknowledgements as below: Fig. 1 and Fig. 4 were produced with the assistance of Servier Medical Art (https://smart.servier.com). No other findings and conclusions in this review are affected by these changes.

The original article has been corrected.

## References

[1] Yang L (2021). The signal pathways and treatment of cytokine storm in COVID-19. Signal Transduct. Target Ther..

